# Sequence-based epitope mapping of high pathogenicity avian influenza H5 clade 2.3.4.4b in Latin America

**DOI:** 10.3389/fvets.2024.1347509

**Published:** 2024-04-29

**Authors:** Andres F. Ospina-Jimenez, Arlen P. Gomez, William F. Osorio-Zambrano, Santiago Alvarez-Munoz, Gloria C. Ramirez-Nieto

**Affiliations:** Microbiology and Epidemiology Research Group, Facultad de Medicina Veterinaria y de Zootecnia, Universidad Nacional de Colombia, Bogotá, Colombia

**Keywords:** antigenic variability, avian influenza, H5N1, South America, HPAI, Latin America

## Abstract

High Pathogenicity Avian Influenza (HPAI) poses a significant threat to public and animal health. Clade 2.3.4.4b recently emerged from the Eastern hemisphere and disseminated globally, reaching the Latin American (LATAM) region in late 2022 for the first time. HPAI in LATAM has resulted in massive mortalities and culling of poultry and wild birds, causing infection in mammals and humans. Despite its meaningful impact in the region, only occasional evidence about the genetic and epitope characteristics of the introduced HPAI is reported. Hence, this study seeks to phylogenetically characterize the molecular features and the source of HPAI in LATAM by evaluating potential antigenic variations. For such a purpose, we analyzed 302 whole genome sequences. All Latin American viruses are descendants of the 2.3.4.4b clade of the European H5N1 subtype. According to genomic constellations deriving from European and American reassortments, the identification of three major subtypes and eight sub-genotypes was achievable. Based on the variation of antigenic motifs at the HA protein in LATAM, we detected three potential antigenic variants, indicating the HA-C group as the dominant variant. This study decidedly contributes to unraveling the origin of the 2.3.4.4b clade in LATAM, its geographic dissemination, and evolutionary dynamics within Latin American countries. These findings offer useful information for public health interventions and surveillance initiatives planned to prevent and manage the transmission of avian influenza viruses.

## 1 Introduction

High Pathogenicity Avian Influenza (HPAI) is a contagious disease that affects poultry, wild birds, and occasionally mammals (1). It has devastating consequences for animal and human health due to its high mortality and zoonotic capacity. HPAI also leads to financial loss because of mass mortality and culling of poultry, as well as international bans on avian products from countries where the disease is present. Avian influenza is caused by certain strains of the Influenza A virus (FLUAV), member of the *Orthomyxoviridae* family, genus *Alphainfluenzavirus* (2). FLUAV is composed of eight negative ssRNA genomic segments that show relevant phylogenetic diversity owing to a high mutation rate and genomic reassortment. FLUAV is classified into subtypes based on the characteristics and combination of the two major surface glycoproteins: Hemagglutinin (HA) and Neuraminidase (NA). Currently, 16 HA and 9 NA types have been described in avian reservoirs of the Charadriiforme and Anseriforme orders (3, 4). These subtypes are commonly described as causing localized respiratory and gastrointestinal infections that lead to Low Pathogenicity Avian Influenza disease (LPAI). Nevertheless, subtypes H5 and H7 have been related to the systemic infection that causes High Pathogenicity Avian Influenza (HPAI). The evolution of LPAI-to-HPAI in both H5 and H7 influenza virus subtypes is the result of a genetic predisposition that allows the gain of multiple basic amino acids at the cleavage site of the immature HA gene (5). As a result, the HA undergoes activation by cellular endoproteases that exist in cells through multiple avian and organ tissues allowing efficient replication of the virus in several organs resulting in a severe systemic disease (6, 7).

Conversion events from LAPI-to-HPAI viruses have occurred multiple times mainly in intensive poultry production systems, where they had been controlled and maintained geographically limited (8, 9). However, this panorama changed in 1996 when the HA of the HPAI strain A/goose/Guangdong/1/1996 reached wild migratory birds, where it has been maintained and disseminated giving rise to the globally distributed goose/Guangdong (Gs/Gd) lineage (10, 11). After the establishment of the Gs/Gd lineage, it has suffered diversification at phylogenetic and subtype levels through drift and shift changes, respectively. This diversity resulted in the determination of 10 phylogenetic clades, many subclades, and the description of certain H5Nx HPAI virus subtypes (12, 13). The Gs/Gd lineage was first reported in America by the end of 2014 when introductions of an Asian H5N8 of the clade 2.3.4.4 were identified in North America. This subtype as well as emergent H5Nx reassortants, spread across the region being detected in wild birds and poultry causing the 2014/2015 epizootic outbreaks (14–16). It is known that the original virus entered America through migratory flyways in the Pacific north (17). Despite the 2014/2015 epizootics in North America, at that time, the HPAI did not extend to Latin America, and it could be controlled and eradicated. Any detection in wild birds in America was reported after 2015 suggesting its disappearance from the continent (18).

The Gs/Gd lineage continued circulating in Africa, Asia, and Europe causing seasonal outbreaks that remained restricted to the Old World. However, the recent 2020–2021 epidemics caused by the 2.3.4.4b clade reached panzootic dimensions being reported in all the continents affecting a wide range of hosts including several mammalian species. Particularly in America, the panzootic clade was introduced two times into North America from two different geographic regions. The first known introduction was caused by the arrival of a Eurasian H5N1 FLUAV (EA) in the United States through transatlantic flyways (19–21). The second entrance was reported in Canada and was originated by an Asian H5N1 that reached the continent through the Bering Strait (21). The arrival of the panzootic HPAI to the continent led to the emergence of new EA/American reassortants, outbreaks in poultry and wild birds, infection in mammals, and for the first time, the spread of the virus into Latin American countries (22, 23). The 2.3.4.4b clade in Latin America was initially reported in October 2021 when the disease was confirmed in backyard poultry and a wild duck (*Spatula discors*) in Colombia. Subsequently, new detections came from pelicans in Venezuela; wild birds, backyard poultry, and sea mammals in Peru; backyard and commercial poultry in Ecuador, Bolivia, and Argentina; wild and domestic birds in Chile; wild birds in Panama, Honduras, Guatemala, and Cuba; and wild birds in Brazil, Uruguay, and Paraguay (24–26). The appearance of HPAI in the Latin American countries resulted in the death and culling of thousands of poultry, massive deaths of sea mammals, and the first human cases of this disease in the region (27, 28).

Currently, some studies have shown the impact of HPAI in Latin America (27) and the molecular characteristics of the HA gene in Ecuador, Peru, and Chile (26, 29, 30). Recently, new reports have been published in Uruguay and Brazil (31, 32). However, research about the molecular origin and evolution of the virus in the region, as well as its antigenicity and genomic configuration remains scarce. Therefore, the objective of this study was to determine the molecular characteristics and origin of the FLUAV in Latin America, describe circulating genotypes, and identify the emergence of potential antigenic variants through epitope characterization. This study proposed strains as representatives of the viral panorama in the region.

## 2 Materials and methods

### 2.1 Sequence selection and phylogenetic analysis

Available sequences of HPAI H5Nx reported between 2021 and 2023 in Latin America were compiled from the global data science initiative – GISAID.^1^ Sequences were filtered down using the following criteria: Hemagglutinin H5 subtype, 2.3.4.4b clade, collected in the Americas region (excluding United States and Canada), collection dates between 01 January 2021 and 12 August 2023, and complete segments for PB2, PB1, HA, NP, NA, M, and NS. This resulted in 100 strains of which 47 were selected considering the phylogenetic relationships that were visualized in a preliminary phylogenetic tree of the HA gene. Three viruses from Uruguay reported after the data collection were also included.

Selected strains were compared to 255 global viruses from America, Europe, Asia, and Africa detected since 1959 encompassing 14 subtypes and multiple clades of the H5 subtype (Supplementary Table S1). Phylogenetic trees for each gene were inferred by the maximum likelihood approach implemented in the IQ-TREE v2.2.2.6 software with branch support of 1,000 ultrafast bootstrap. Trees were visualized and edited with the Interactive Tree Of Life (iTOL)^2^ v6.7.5. Based on the topology of map introductions, genotypes (GT) and sub-genotypes (SGT) were inferred. Latin American introductions were defined when new viruses or phylogenetic groups appeared on the trees, while GTs and SGTs were determined according to the combination of subclades in the eight genomic segments. To identify the probable American FLUAVs that contributed to the genomic constellations observed, a BLAST search was carried out using the BLAST tool of the NCBI (accessed on 13 November 2023). For this analysis, one strain of the main GTs and SGTs was selected as a reference for the search.

### 2.2 Sequence-based mapping of HA

The variation of the HA and its epitopes (equivalent to A and B of H3 and Sa of H1) was evaluated by the sequence-based mapping linear approach developed by Anderson et al. (33). For this purpose, amino acid sequences were downloaded from the GISAID database (accessed on 25 August 2023), aligned by the MUSCLE-5 method, and edited to obtain proteins in the H3 numbering. For comparing epitopes, amino acids were extracted in the Spyder integrated development environment (Python 3.11) using the biopython package. All analyses were carried out based on Hamming Distance calculations using the *stringdist* package v0.9.10 implemented in the RStudio^®^ software v4.3.1. A hierarchical cluster analysis was also performed to visualize epitope groups and to select potential representative strains. The number of clusters to be represented was selected according to the topology of the maps.

## 3 Results

### 3.1 European H5 2.3.4.4b clade has been introduced into Latin America at least eight times and N1 six times giving rise to new subclades in Central and South America

**Figure 1:**
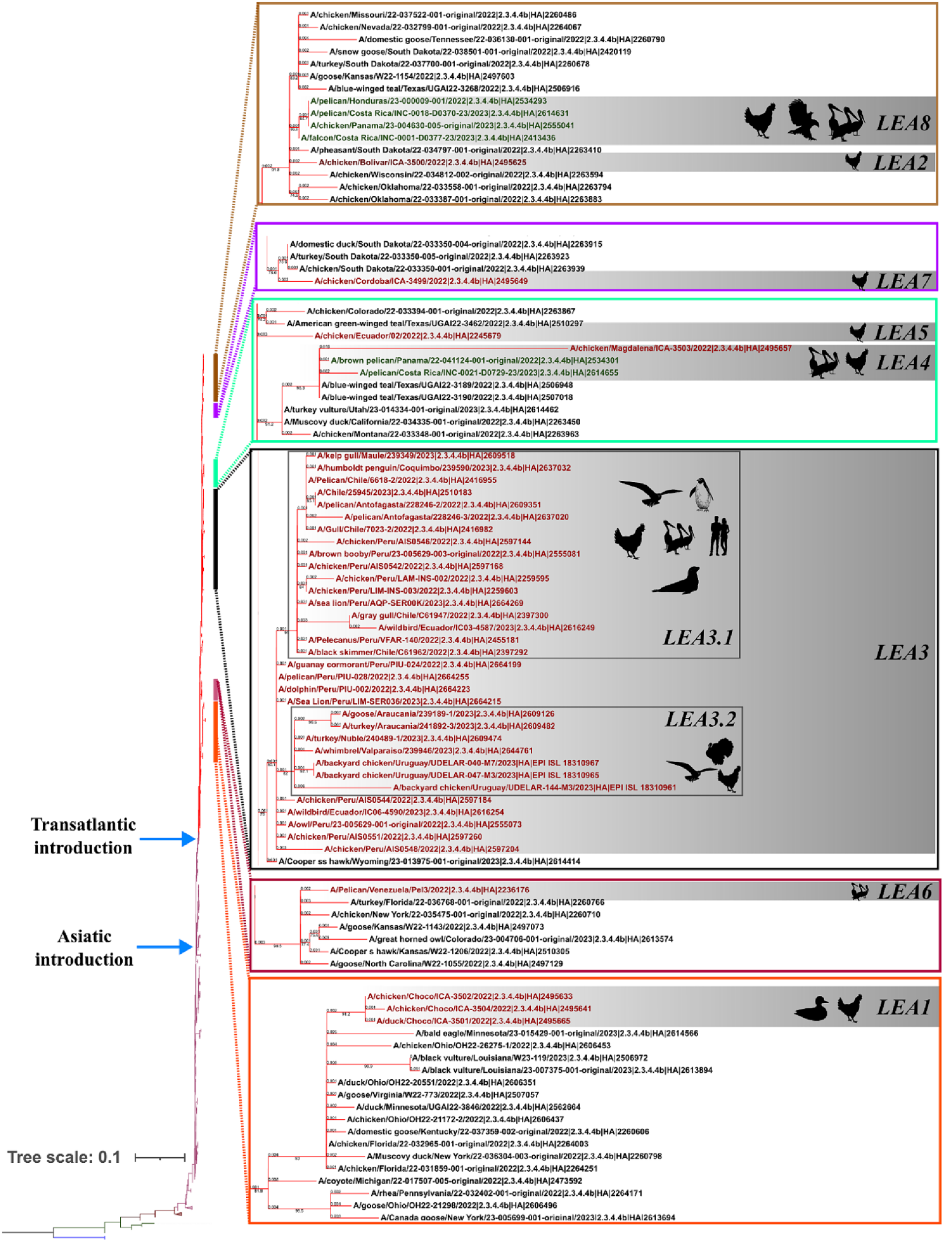
Phylogenetic tree of the HA gene and species where it has been detected. Branches are colored according to their phylogeny. Blue: American H5; Green: European Non-goose/Guangdong HPAI. Brown: Goose/Guangdong Lineage; Purple: 2.3.4.4b clade; American 2.3.4.4b subclade. Latin American strains labels are colored according to the region where they were detected. Red: South America; Green: Central America. Latin American subclades were named according to the lineage and chronological order in which they were detected, and they are shown in gray boxes. LEA stands for: Latin American – Eurasian Lineage.

**Figure 2:**
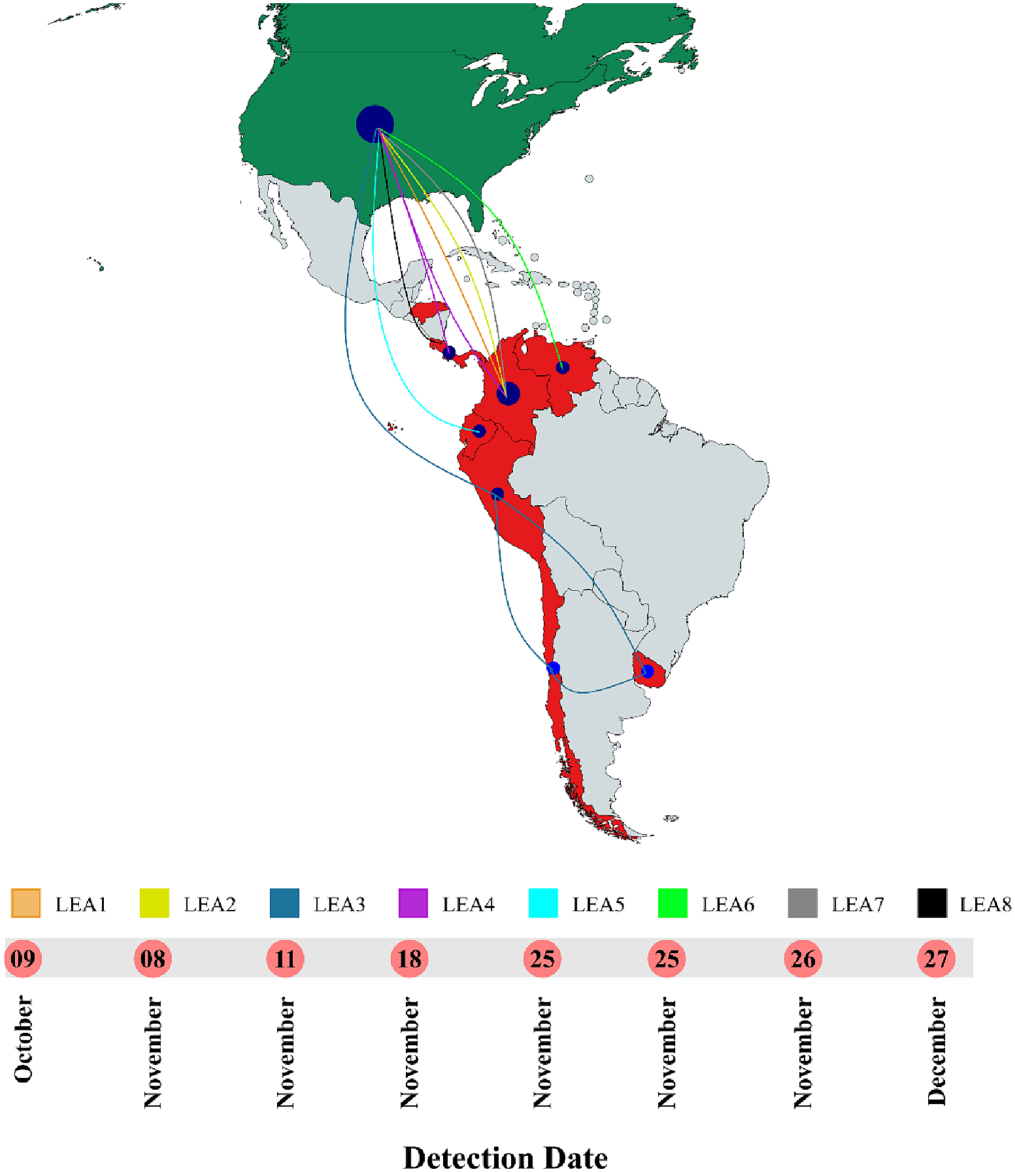
Timeline and map of introductions of HPAI in Latin America. All entrances were detected in 2022. LEA stands for: Latin American – Eurasian Lineage. Map was created with mapchart.net.

Phylogenetic analysis indicated that the H5 2.3.4.4b clade had been introduced into Latin America at least eight times since 2022 and that all viruses that reached the region were descendants of the European strains that arrived at the continent through the transatlantic route (Figure 1 and Supplementary Figure S1). Those were named as Latin American Eurasian descendants (LEA). Chronologically, the first viruses were identified in the Choco region in Colombia located in the northwest of South America in the Atlantic Ocean border. They appeared in October 2022, and they showed some phylogenetic variation forming a monophyletic group that was identified in poultry and a specimen of *Spatula discors* only in that country (LEA1). LEA1 viruses were located apart from other South American viruses, and they were grouped to strains from Florida at the Southeast of the USA. The second introduction (LEA2) was also reported in Colombia and appeared in November 2022 as a single branch in the phylogenetic tree that was related to strains from the Midwest region of the USA. The third incursion occurred in Peru also in November 2022 and it was the most relevant due to their wave-like spread in the region reaching Chile, Ecuador, and Uruguay. The spread resulted in the evolution of a monophyletic branch (LEA3) that was associated with a strain from Wyoming, USA. In this group, the mammal-infecting viruses including the human case from Chile (A/Chile/25945/2023) were allocated. LEA3 showed evolution into two subclades: one including viruses from Peru, Chile, and Ecuador (LEA3.1), and another including viruses from Chile and Uruguay (LEA3.2). The fourth entry was first detected in Colombia, and it originated from the same viral pool described in Texas, that gave origin to viruses detected later in Central America. For that reason, those were considered as a single introduction (LEA4). The fifth entrance took place in Ecuador and was visualized as a single branch that was related to viruses from Texas and Colorado in the USA (LEA5). The sixth introduction occurred in Venezuela and was related to viruses from the East region of the USA (LEA6). The seventh arrival was again detected in Colombia, and it was associated with strains from South Dakota, USA (LEA7). The eighth incursion was detected in Central America and occurred in Panama, Honduras, and Costa Rica (LEA8). This subclade was associated with a Colombian H5 virus detected earlier during the second dissemination of the clade in Latin America and to viruses from the Midwest of the USA. A map that summarizes incursions of HPAI in the Latin American region could be observed in Figure 2.

**Figure 3:**
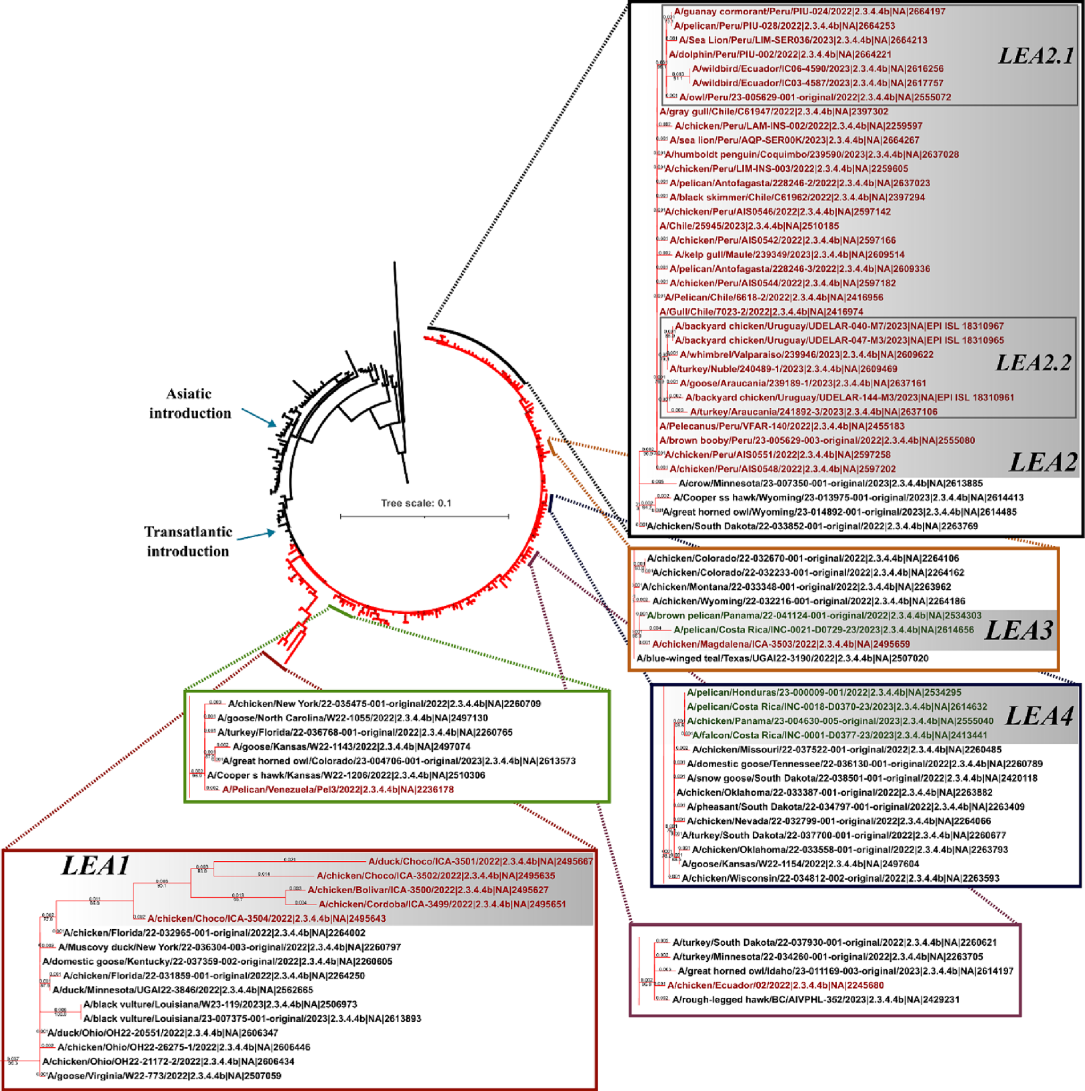
Phylogenetic tree of NA gene. Branches of American viruses are colored in red. Latin American strains are colored according to their geographic origin. Red: South America; Green: Central America. Subclades were named according to the lineage and chronological order of their detection and are shown in gray shadows. LEA stands for: Latin American – Eurasian Lineage.

In line with observations made for the HA gene, the phylogeny of the NA indicated that all viruses in Latin America carried genes derived from the European FLUAVs (Figure 3 and Supplementary Figure S1). However, it revealed only six independent introductions as well as the evolution of the gene into four subclades, suggesting shared circulation of phylogenetic-related NA genes in Latin American strains. The first subclade in the region corresponded to Colombian strains (LEA1). This subclade showed a high similarity with viruses from Florida and was formed by viruses from the first introduction into the Choco region and subsequent introductions in the same country (Figure 3 and Supplementary Figure S2). The second subclade was formed by FLUAVs that arrived at Peru during the third incursion of the clade into South America and gave rise to a monophyletic subclade (LEA2). LEA2 was present in Ecuador, Chile, and Uruguay. Topology of LEA2 agreed with the observation made in the LEA3 group of the HA regarding its evolution into two subclades: one encompassing Peruvian and Ecuadorian strains (LEA2.1) and another including viruses from Peru, Chile, and Uruguay (LEA2.2). A third phylogenetic group was composed of viruses from Colombia and Central America (LEA3), which corresponded to the fourth incursion of HPAI viruses related to strains from Texas, Wyoming, Colorado, and Montana, USA. The fourth identified NA group was a Central American monophyletic group related to strains from the South-center of the USA that was formed by viruses from Panama, Honduras, and Costa Rica (LEA4). In addition, two viruses located as single branches were observed for the Venezuelan virus which exhibited similarity with a strain from Kansas and an Ecuadorian virus that was related to viruses from the North of the USA and Canada (Other LEAs).

### 3.2 Latin American H5N1 viruses showed three main genotypes and at least eight sub-genotypes that carried American and Eurasian genes

**Figure 4:**
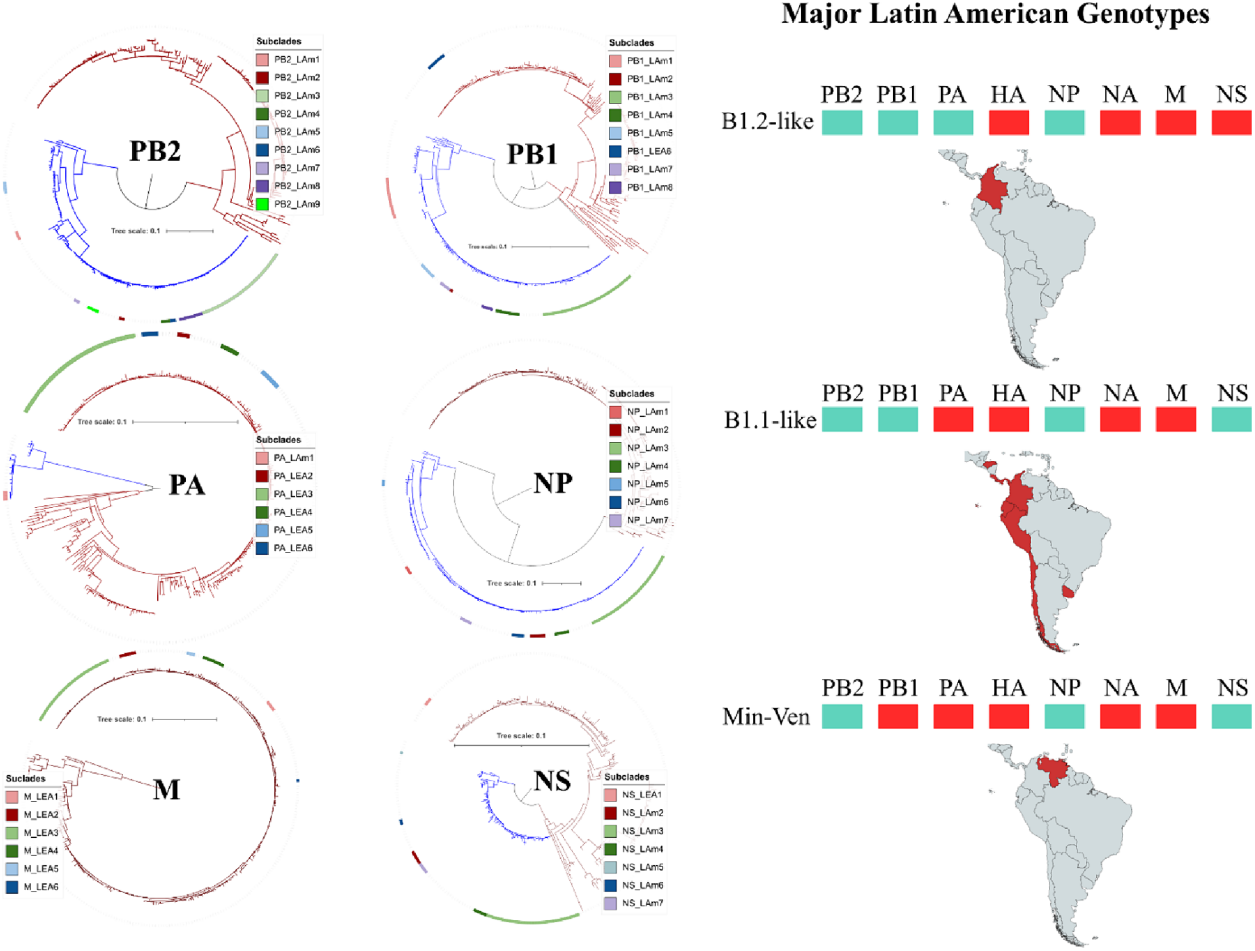
Phylogenetic trees of internal genes major subtypes, and their geographic distribution in Latin America. Branches and boxes are colored according to the major lineage. Red: Euro-Asian; Cian: American. Subclades were named according to their lineage and chronological detection. Maps were created with mapchart.net.

The genomic pools of Latin American H5N1 HPAI viruses were dominated by descendants of FLUAVs previously reported in the American region (AM lineage) in the PB2, PB1, NP, and NS genes and by the EA region (EA lineage) in the PA and M genes (Figure 4 and Supplementary Figure S2). In the PB2 tree, all analyzed viruses were allocated in the AM lineage and distributed into nine subclades (LAm1-9). The greatest portion of the strains was positioned in the subclade that contained viruses from the incursion of the HPAI virus into Peru (PB2_LAm3). In PB1, viruses were distributed into six AM (PB1LAm1-5, and 7) and one EA subclade (PB1_LEA6). In this segment, PB1_LAm3 and PB1_LAm8 were notorious for being the biggest subclades containing strains from Peru and other countries from South America, and the Central American region, respectively. The only PB1 EA gene was found in the virus A/Pelican/Venezuela/Pel3/2022. In the PA segment, five subclades corresponded to the EA (PA_LEA2-6) and one to the AM lineage (PA_LAm1). Like the observations with the other polymerase genes, most viruses were positioned in the clade that corresponded to the third intracontinental movement of the HPAI virus (PA_LEA3). The PA_LAm1 was present in viruses from Choco, Colombia. Regarding NP, with no exception, all viruses carried an AM gene and were distributed into nine subclades with the Peruvian subclade showing dominance (NP_LAm3). In the Matrix gene, the EA lineage was consistently identified in all Latin American viruses. Those were dispersed into six subclades (M_LEA1-6), of which the third (M_LEA3) was also the dominant. For NS, most viruses were allocated into six AM subclades (NS_LAm2-7). The only exceptions were viruses from Choco, Colombia that carry EA genes of the NS_LEA1 subclade.

Based on the major lineage and the combination of subclades, three GTs and at least eight SGTs were identified in Latin America (Figure 4 and Supplementary Table S2). The first was represented by the strain A/duck/Choco/ICA-3501/2022, which had a genomic constellation like the B1.2 GT reported in North America; therefore, it was named B1.2-like (22). This GT was the first introduced into Latin America and it was only detected affecting backyard poultry and a wild duck in Colombia. B1.2-like harbored AM PB2, PB1, PA, and NP of the first Latin American subclades (LAm1) in combination with EA HA, NA, M, and NS of the first subclades (LEA1). The GT was frequently associated with North American strains from Florida and Ohio (Figures 1, 2 and Supplementary Figures S1, S2). According to the BLAST analysis, AM viruses that could contribute to the emergence of this GT were: A/Mallard/Alberta/175/2021 (H4N6) (98.62% identity in PB2), A/Mallard/Alberta/357/2022(H3N8) (99.53% identity in PB1), A/mallard/Minnesota/UGAI18-1849/2018(H4N6) (98.81% identity in PA), and A/Mallard/Alberta/597/2021(H2N4) (99.03% identity in NP) (Figure 4).

The second Latin American GT (B1.1-like) was like the B1.1 also described in North America (22). The genomic constellation in this GT was composed of EA PA, HA, NA, and M in combination with AM PB2, PB1, NP, and NS. This GT had the widest geographic distribution, and it was the most diverse, accounting for eight SGTs. It was first identified in Colombia during the incursion of HPAI virus into backyard poultry in the Bolivar region in the North of South America (SGT B1.1-like-a). However, it was not established in South America until the independent introduction of a new strain into Peru. Once in the region, the GT spread into Chile, Ecuador, and Uruguay (SGT B1.1-like-b). Afterwards, this GT was again independently introduced into Colombia and Ecuador as well as in Panama, Honduras, and Costa Rica resulting in the relevant SGT diversity observed (SGT B1.1-like-c-h). The most prevalent SGTs were the B1.1-like-b which were widespread in South America, and the B1.1-like-g which dominated the panorama in Central America.

**Figure 5:**
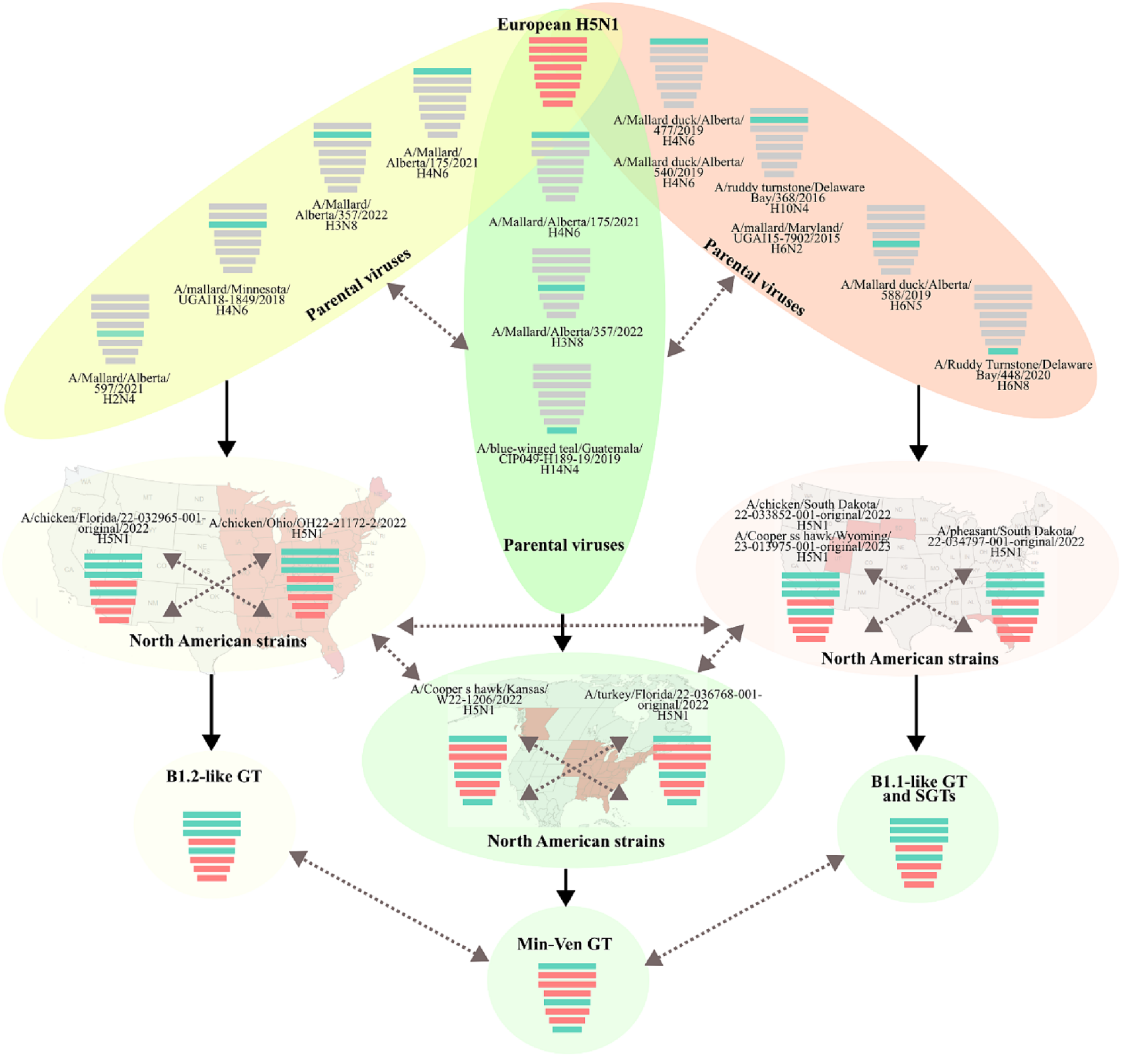
Gene flow and probable origin of H5N1 Genotypes and Sub-Genotypes identified in Latin America. The color of genomic segments indicates its lineage, Cian: American; Red: Eurasian. Parental and North American strains correspond to viruses that were related to the representative strains of Latin American Genotypes. The geographic distribution of North American strains is showed in the background. Solid arrows indicate the probable gene flow that gave rise to the genomic constellations observed in the region. Discontinued arrows highlight possible unknown gene movements. Maps were created with mapchart.net.

The Peruvian B1.1-like-b SGT was represented by the strain A/owl/Peru/23-005629-001-original/2022. This SGT was a fully newly introduced virus that harbored new genomic subclades in all genes (LAm3, LEA3 subclades in HA and internal genes, and LEA2 in NA). Those subclades were associated with North American viruses (Figure 5). According to BLAST analysis, the probable AM donors of segments PB2, PB1, NP, and NS were A/Mallard duck/Alberta/477/2019(H4N6), A/Ruddy turnstone/Delaware Bay/368/2016(H10N4), A/Mallard duck/Alberta/588/2019(H6N5), and A/Ruddy Turnstone/Delaware Bay/448/2020(H6N8) which showed 99.29, 98.58, 98.20, and 99.16% identity in the PB2, PB1, NP, and NS genes, respectively.

Regarding B1.1-like-g SGT, it was first detected in Honduras during the eighth introduction of the virus in Latin America and was represented by the virus A/pelican/Honduras/23-000009-001/2022. The SGT showed similarity in most of the genomic segments with viruses from South Dakota and exhibiting relatedness in PA with a strain from Nevada. Its PB2, PB1, HA, M, and NS genes were originated from the same pool of ancestors of the Colombian strain A/chicken/Bolivar/ICA-3500/2022, however, it evolved forming an independent monophyletic subclade. Conversely, its PA, NA, and NP genes were only associated with North American FLUAVs. The probable donors of AM genes in this SGT were A/Mallard duck/Alberta/540/2019(H4N6), A/Mallard/Maryland/UGAI15-7902/2015(H6N2), A/Mallard duck/Alberta/588/2019(H6N5), and A/Ruddy Turnstone/Delaware Bay/448/2020(H6N8) exhibiting a 99.09% identity in PB2, 98.38% in PB1, 98.80% in NP, and 98.93% in NS.

The final GT corresponded to the newly introduced virus A/Pelican/Venezuela/Pel3/2022 that reached Venezuela and was composed of PB2, NP, and NS from the AM lineage and PB1, PA, HA, NA, and M of the EA lineage. It was associated with viruses from Kansas, New York, Florida, and Canada. This GT had been reported as a minor group in North America and was named Min-Ven GT (22). In contrast to other GTs, the probable donors of AM genes of the Min-Ven were traced to two strains from Canada: A/Mallard/Alberta/175/2021(H4N6) (98.77% identity in PB2) and A/Mallard/Alberta/357/2022(H3N8) (98.90% in NP), and the A/blue-winged teal/Guatemala/CIP049-H189-19/2019(H14N4) from Central America (98.98% identity in NS). The GTs and SGTs that harbored Latin American viruses analyzed in this study are summarized in Supplementary Table S2.

### 3.3 Sequence-based mapping of Latin American H5 HPAI viruses showed variation in the epitopes of the HA and suggest the frequent introduction and the emergence of potential antigenic variants

**Figure 6:**
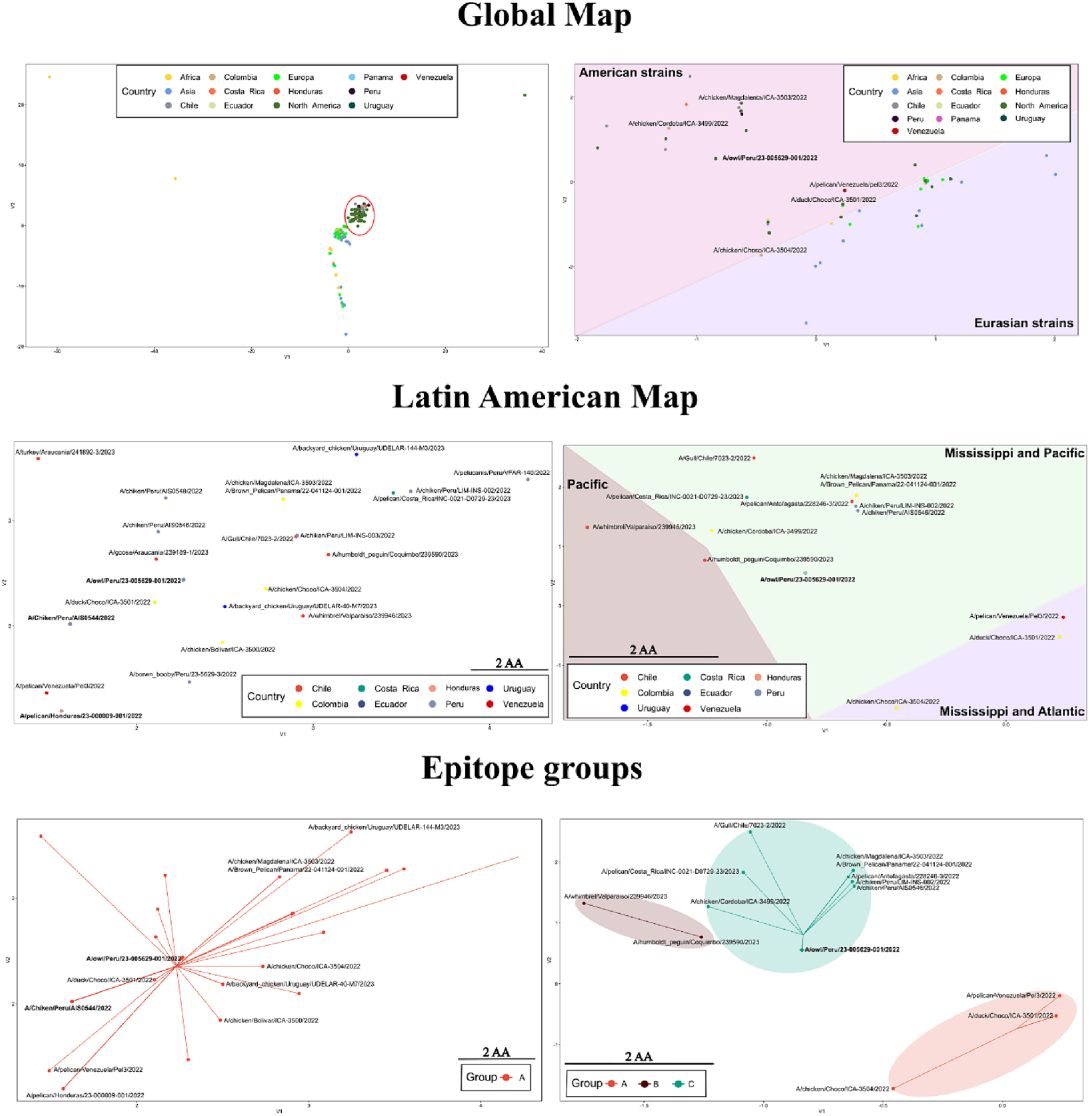
Variation in sequence-based maps on whole-protein (left) and epitope (right) of HPAI H5 2.3.3.4b. Labels in bold indicate positions in the maps where many Latin American strains were allocated. Red circle highlights the position of Latin American viruses in the whole-protein global map. For the construction of the maps only 2.3.4.4b strains were considered.

Based on the global map of the whole protein, viruses of the H5 2.3.4.4b clade showed a strong relatedness and tendency to be placed together (Figure 6 and Supplementary Table S3). In this map, a lineal-like tendency was observed indicating the evolution of the HA into a major subgroup in America from European strains. According to mutations in the whole protein, the variation could be represented into two subgroups (HA-A and HA-B; Supplementary Figure S3). Latin American FLUAVs were like the North American viruses since both were found to intermix. When zooming in to those viruses, some level of variation is appreciated with a mean difference of 2.9 ± 1.7 amino acids (0–8/562). However, it does not appear to be related to geographic patterns, being viruses from Central and South America placed near to each other. In addition, all included viruses were assigned to the same protein subgroup (HA-A). The pattern in the map suggested the spread and dominance of three variants of the HA gene in the region. Those were the A/Chicken/Peru/AIS0544/2022-like (A/ck/Peru/544-like), A/owl/Peru/23-005629-001/2022-like (A/owl/Peru/629-like), and A/pelican/Honduras/23-000009-001/2022 (A/pel/Honduras/09-like). The A/ck/Peru/544-like was present in viruses from Peru, Ecuador, and Chile including the human strain; the A/owl/Peru/629-like HA in strains from Peru, Colombia, and Ecuador, and the A/pel/Honduras/09-like in viruses from Panama, Costa Rica, and Honduras.

When only the epitope regions of the 2.3.4.4b clade were considered, the global map showed a dispersal distribution that better reflected the influence of geographical factors. In that map, Latin American and certain North American viruses were positioned apart from European, African, Asian, and other North American viruses (Figure 6). The global antigenic profile diversity in HA could be represented in four subgroups (HA-A, HA-B, HA-C, HA-D, Supplementary Figure S3). Most of the Latin American viruses were allocated to the HA-C subgroup. However, certain strains were assigned to the HA-A and HA-B. In contrast to what was seen in the whole protein maps, the epitope variation suggested the existence of a single dominant variant in Latin America as most of the viruses (67.3%) were represented as a single point that corresponded to the position of the A/owl/Peru/23-005629-001/2022 virus (A/owl/Peru/629-like), which showed an epitope identity above 95% with 41 of the Latin American FLUAVs.

## 4 Discussion

This research contributes to unraveling the intricate web of viral introductions, geographic dissemination, and evolutionary dynamics of avian influenza virus, shaping the landscape of the European 2.3.4.4b clade within Latin American countries. The identification of distinct geographic associations provides pivotal insights, crucial for devising strategies to curb the spread of this clade in the region. These findings offer valuable information which can be used in future public health interventions and surveillance initiatives aimed on preventing and managing the transmission of avian influenza viruses.

Despite the transmission of H5 avian influenza viruses between the Eastern and Western Hemispheres has been infrequent and appeared to be mainly restricted to intercontinental flyways through the Pacific coast, it was notorious that the current FLUAVs in LATAM were originated from European strains which reached the continent through transatlantic flyways. Considering that the most of incursions identified in this study primarily occurred along the Pacific-Central flyway (LEA1-5 and LEA7-8), it was noted that the appearances in North American west coast were a critical step for the introduction of HPAI in LATAM, as it has been demonstrated for other subtypes (34). The need for this east-to-west expansion of the European 2.3.4.4b could explain the phylogenetic diversity observed among the introductions detected in LATAM. We consider that this diversity is the result of the ongoing evolutionary dynamics suffered through drift and shift of the clade during its dissemination within wild birds in North America, considered the main source of novel genetic and genotype variants in Central and South America.

Among evaluated incursions of the 2.3.4.4b clade in LATAM, the most relevant was the Peruvian which resulted in the establishment of a widespread South American clade named LEA3. This clade has been described affecting a wide spectrum of animal populations and causing the first known human case in the region. The emergence of LEA3 in the continent reflects a combination of environmental, human-induced, and potentially adaptative viral factors that supported the conformation of an ecological “niche” where the virus remains in circulation and expands to surrounding countries like Chile, Ecuador, and Uruguay. The role of Peru in the dissemination of the 2.3.4.4b clade in LATAM was configured by their geographic location, as this country is crossed by the Pacific flyway which serves as a natural highway for the expansion of FLUAVs in South America. It was also notorious that among LATAM countries, Colombia had the higher count of introductions. This could be the result of its unique geographical location and features which makes Colombia a hotspot for the entrance of North American viruses. Among these features are the presence of the Pacific and Mississippi migratory flyways, dual coastlines, diverse ecosystems, a tropical climate, and its abundant biodiversity (35–37). However, it was remarkable that all Colombian entrances showed a restricted circulation. This situation could be the result of the implementation of immediate control measures and eradication programs that, along with viral factors, limited its spread and infectivity. In addition, the existence of climate and natural barriers in the Colombian geography may have played a role in this situation.

LATAM strains in this study portray a complex genomic landscape resulting in three major GTs, which displayed a diverse constellation of genes from AM and EA origin derived from both HPAI and LPAI FLUAVs. This intricate genetic makeup demonstrates the impact of the introduction of novel genes in the AM viral pool and this is probably the explanation of the epidemiological characteristics of the 2.3.4.4b clade in LATAM. It is likely that the gain of LPAI-derived genes adapted to Latin American hosts accounted for the increased infectivity of the virus in mammals and its tropism for American pelicans. However, this must be evaluated in detail through *in vivo* assays and further analysis.

An in-depth analysis across all GTs unveils a significant clustering of the PB2 gene of LATAM HPAI within the AM lineage. This suggest that the gain of an AM PB2 played a pivotal role in the emergence of 2.3.4.4b FLUAVs in LATAM. Since it is known that PB2 is a major key in host adaptation, we consider that the gain of an AM PB2 offered an evolutive advantage for the reassorted FLUAVs allowing their spread in Central and South America. This appeared to be also the case for NS and PB1, which are related to avoid the antiviral response and polymerase activity, respectively. In these genes, an AM origin was detected in the widespread GT 1.1-like, suggesting that, viruses carrying them have an improved fitness in American hosts than GTs 1.2-like and Min-Ven. This could explain the limited circulation of B1.2-like and Min-Ven among the LATAM region.

The whole protein analysis underscored intriguing patterns within the 2.3.4.4b clade, notably showcasing a tendency to cluster indicating a relative low variation. This phenomenon delineated a linear evolutionary trajectory, providing evidence for the emergence of a global predominant subgroup of the HA gene in the Americas originated from European strains. Upon scrutinizing LATAM and North American FLUAVs a substantial similarity was apparent with a notable intermixing pattern in the maps. However, a closer inspection revealed subtle variations within these viruses, which intriguingly did not correspond to discernible geographic patterns. Despite this, viruses from Central and South America exhibited a proximity in the mapping. Based on these analyses, the prevalence and dissemination of three dominant variants of the HA gene within LATAM were identified. These variants were represented by A/Chicken/Peru/AIS0544/2022-like (A/ck/Peru/544-like), A/Owl/Peru/23-005629-001/2022-like (A/owl/Peru/629-like), and A/Pelican/Honduras/23-000009-001/2022 (A/pel/Honduras/09-like) variants. A/ck/Peru/544-like and A/pel/Honduras/09-like were the most relevant as they dominated the panorama in South and Central America, respectively. Considering that A/owl/Peru/629-like and A/pel/Honduras/09-like encompassed FLUAVs from the phylogenetic groups LEA3 and LEA8, it is likely that mutations in the HA that gave rise to these variants occurred in LATAM during the outbreaks in wild and domestic birds.

In contrast to what was seen in whole protein, investigation of antigenic motifs highlighted a greater variation, resulting in a geographic-dependent distribution pattern. In these analyses, LATAM viruses, alongside certain North American strains, appeared distinctly separate from European, African, Asian, and additional North American viruses. Most LATAM viruses were clustered within the HA-C subgroup which was also represented by the strain A/Owl/Peru/23-005629-001/2022. These observations suggest that antigenic motifs in the 2.3.4.4b clade have evolved differently after its introduction in the Americas, and that this divergence has been influenced by migratory flyways. For instance, viruses originating from countries within the Mississippi and Atlantic flyways, such as Colombia and Venezuela, were predominantly grouped under HA-A. In contrast, viruses from regions along the Mississippi and Pacific flyways (Central America, Colombia, Peru, Ecuador, Chile, and Uruguay) were positioned under HA-C. In addition, the HA-B subgroup encompassed viruses detected solely in regions associated with the Pacific flyway.

Vaccination against Highly Pathogenic Avian Influenza (HPAI) in poultry has been a key aspect of disease management strategies in some countries. While stamping out remains the preferred method for eradicating outbreaks, resource limitations have led several countries to adopt vaccination as an additional measure. However, historical limitations have been observed, particularly regarding the evolution of the virus and the efficacy of vaccines.

In Mexico, for instance, studies have shown benefits from vaccination, such as reduced mortality and viral shedding. Yet, challenges persist leading to reduced vaccine efficacy (38–40). Continuous monitoring and molecular characterization are crucial for adapting vaccination strategies. In Colombia, where HPAI outbreaks affect wild birds and backyard poultry, a vaccination program has been proposed, although the application has not yet begun. The evolving nature of avian influenza viruses requires tailored vaccines, as historical vaccines have shown reduced efficacy due to rapid viral evolution. The global impact of the H5N1 2.3.4.4b clade of HPAIV has prompted a reevaluation of vaccination strategies, emphasizing the need for genetically and antigenically relevant vaccines. The potential spread of HPAI to new regions via migratory birds highlights the necessity for a coordinated global approach to vaccination planning and execution. Mismatched vaccines can exert immune pressure, leading to genetic and antigenic changes in circulating viruses, posing challenges to bird populations. Thus, the development and testing of efficient vaccine candidates are essential to combat the spread of avian influenza effectively.

Based on whole protein and antigenic motif analysis in our study, we consider that strains with features like A/Owl/Peru/23-005629-001/2022 could be a relevant candidate for the development of tailored vaccines and diagnostic assays in the region. Nevertheless, this must be validated and compared through antigenic cartography and *in vivo* challenging assays.

## 5 Conclusion

The intricate relationship observed between the introductions of the European 2.3.4.4b clade in Latin America and FLUAVs of various North American regions underscores the significance of understanding avian migratory pathways in the Americas. Furthermore, it is imperative to assess the impact of Asian FLUAVs that have reached North America via the Beringia strait in the virological dynamics in the Continent and to evaluate the risk of their introduction into LATAM. This insight will aid to the recognition of the interconnectedness of avian populations and accentuates the necessity for collaborative efforts and enhanced surveillance to prevent and manage the transmission of avian influenza strains in both North and Latin America effectively. Even though this investigation focused on the H5 HPAI virus in LATAM, it is important to highlight the significant role of LPAI viruses as gene donors. As was demonstrated, AM LPAI viruses contributed to the emergence of viral combinations encountered in LATAM, thereby increasing the diversity and complexity of circulating viruses among various species. This increase of viral diversity enhances the risk of interspecies transmission and the emergence of viruses with pandemic potential, which is particularly critical in the LATAM context given the limited knowledge about LPAI also.

We recognize limitations of this type of analysis such as variations in the HA gene that could occur as a result of isolation and passage procedures in laboratory and that during sequence selection sample source origin was not considered as a strict criteria. However, this was a consequence of limited metadata among published sequences in the GISAID database, making the accurate selection of original material unviable and reducing considerably the information. Therefore, it is necessary to encourage the scientific community to share information to have enough data allowing more comprehensive studies.

In conclusion, in this study we report and evaluate viral dynamics that led to the emergence and spread for the first time of European HPAI in LATAM contributing to the knowledge of evolution and diversity of the virus in the region and the rest of the world. All viruses detected in the eight North-to-LATAM incursions described here were descendants from European FLUAVs that reached the continent thought transatlantic flyways. These viruses spread to the west coast evolving through drift and shift changes acquiring American genes from LPAI FLUAVs and novel antigenic motif constitutions. During this expansion, viruses occasionally reached LATAM through the Atlantic and Mississippi flyways. Nevertheless, the panorama changed when FLUAVs reached the Pacific flyway which represent a pivotal event in the dissemination of HPAI in LATAM. All these factors, contributed to the establishment of a HPAI FLUAV that showed phylogenetic and antigenic characteristics for specific viruses of the region. This highlights the need to conduct surveillance along to studies aimed to know these characteristics of FLUAVs circulating in LATAM.

## Data availability statement

The datasets presented in this study can be found in online repositories. The names of the repository/repositories and accession number(s) can be found in the article/Supplementary material.

## Author contributions

AO-J: Conceptualization, Formal analysis, Writing – original draft, Writing – review & editing, Data curation, Methodology, Software. AG: Formal analysis, Writing – original draft, Writing – review & editing. WO-Z: Formal analysis, Writing – review & editing. SA-M: Formal analysis, Writing – review & editing, Writing – original draft. GR-N: Formal analysis, Writing – original draft, Writing – review & editing, Conceptualization, Project administration, Supervision.

## Footnotes

^1^www.gisaid.org, accessed on 12 August 2023.

^2^ http://itol.embl.de

## Supplementary material

SUPPLEMENTARY DATA SHEET 1 Phylogenetic trees of HA and NA genes. Labels in bold highlight strains of the panzootic outbreak detected in America. Name case in green correspond to genes from Central American and in red to genes from South American strains. Branches are colored according to their HA phylogenetic origin. Blue: American; Green: European Non-goose/Guangdong HPAI. Brown: Goose/Guangdong Lineage; Purple: 2.3.4.4b clade; Red: American 2.3.4.4b sub-clade. For NA, red branches correspond to genes from strains of the H5N1 2.3.4.4b clade detected in America. The proposed subclades into the clades are shown.

SUPPLEMENTARY DATA SHEET 2 Phylogenetic trees of internal genes. Branches are colored according to their phylogeny. Blue: American; Red: Eurasian. Labels in bold highlight strains detected in America. Viruses labeled in green and red came from Central and South America, respectively. The proposed sub-clades into the clades are shown.

SUPPLEMENTARY DATA SHEET 3 Sequence-based whole-protein and epitope maps. Predicted clusters based on each analysis are shown.

SUPPLEMENTARY DATA SHEET 4 Clustering analyses according to epitope variation. Predicted clusters are shown.

SUPPLEMENTARY DATA SHEET 5 Whole-protein and epitope cluster assignment of viruses evaluated in this study.

SUPPLEMENTARY TABLE S1 Strains and metadata used in this study.

SUPPLEMENTARY TABLE S2 Genotype and Sub-genotype assignment of Latin American strains.

SUPPLEMENTARY TABLE S3 Amino acid variation among epitopes of the HA in the predicted three clusters. HA-C was considered as representative.
