# Supplementary figures and images for: Sequence-based epitope mapping of high pathogenicity avian influenza H5 clade 2.3.4.4b in Latin America
Source: Front Vet Sci. 2024 Apr 29;11:1347509. doi: 10.3389/fvets.2024.1347509 (PMC11091830; doi:10.3389/fvets.2024.1347509)

# Whole Protein

Group • A

clusters\_cut • A • B

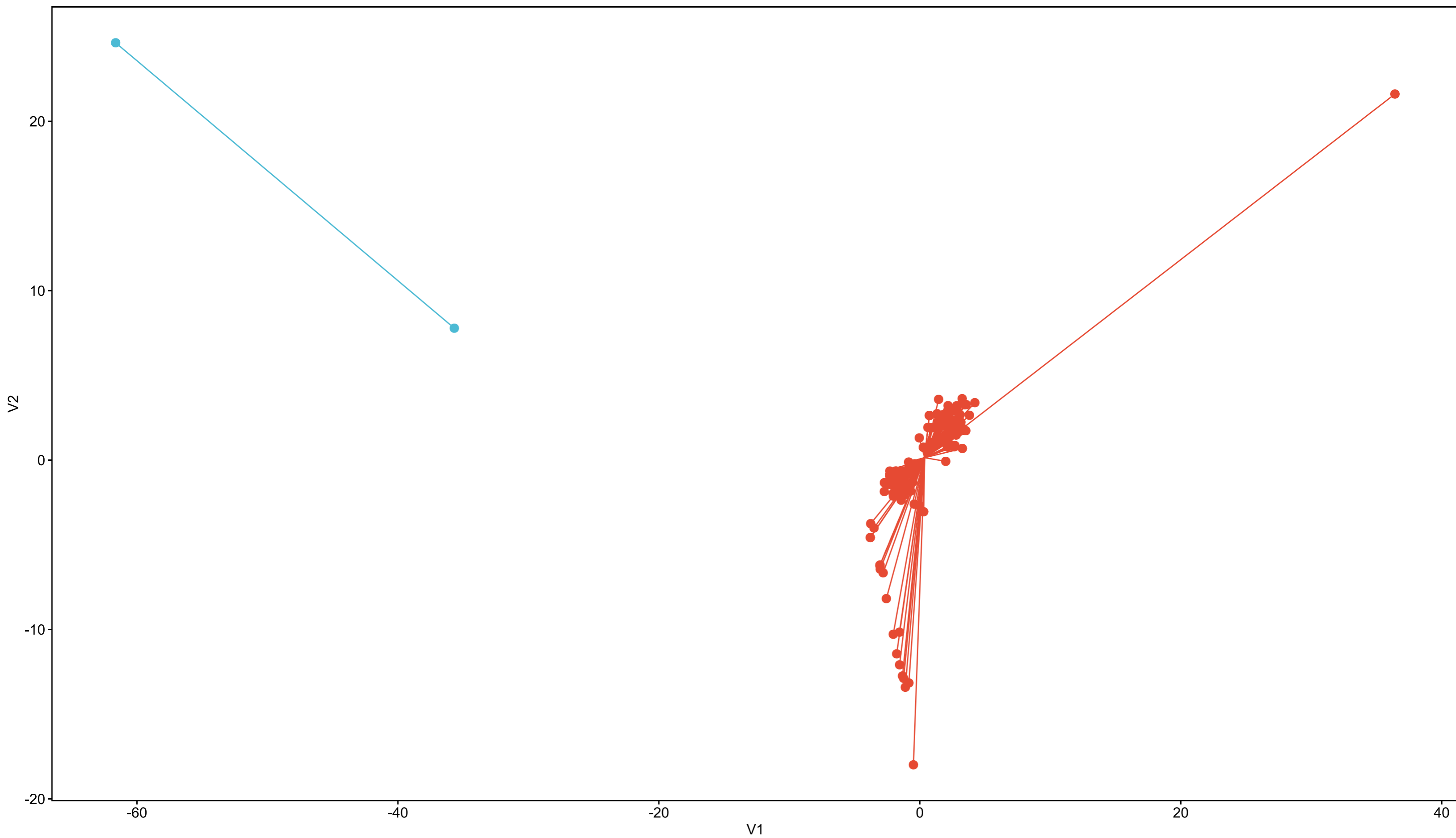

# Epitopes

clusters\_cut • A • B • C • D

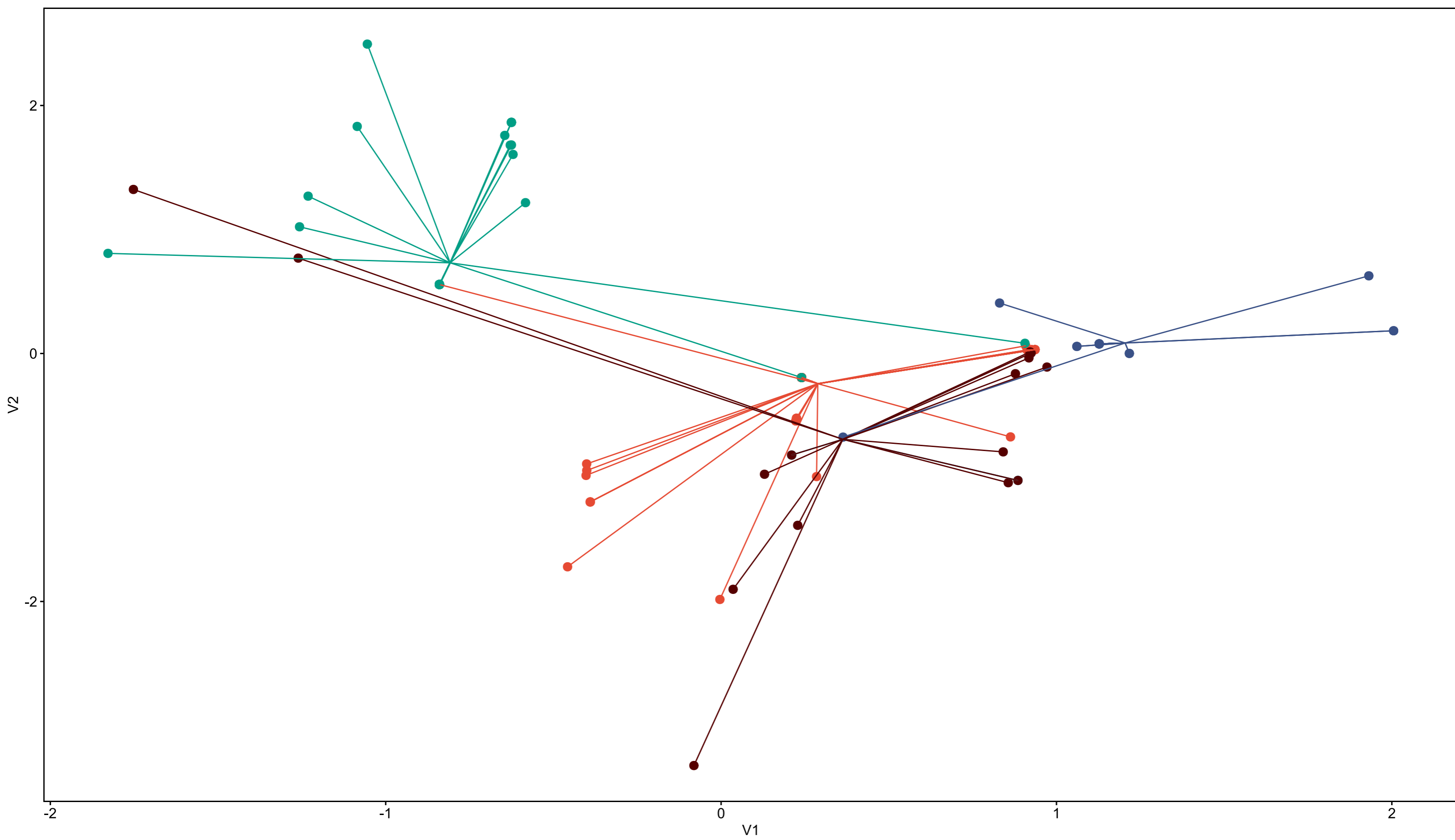

Supplement: SUPPLEMENTARY DATA SHEET 3 — Sequence-based whole-protein and epitope maps. Predicted clusters based on each analysis are shown. [file Data_Sheet_3.PDF]

```
distance_matrix
hclust (*, "ward.D")
```

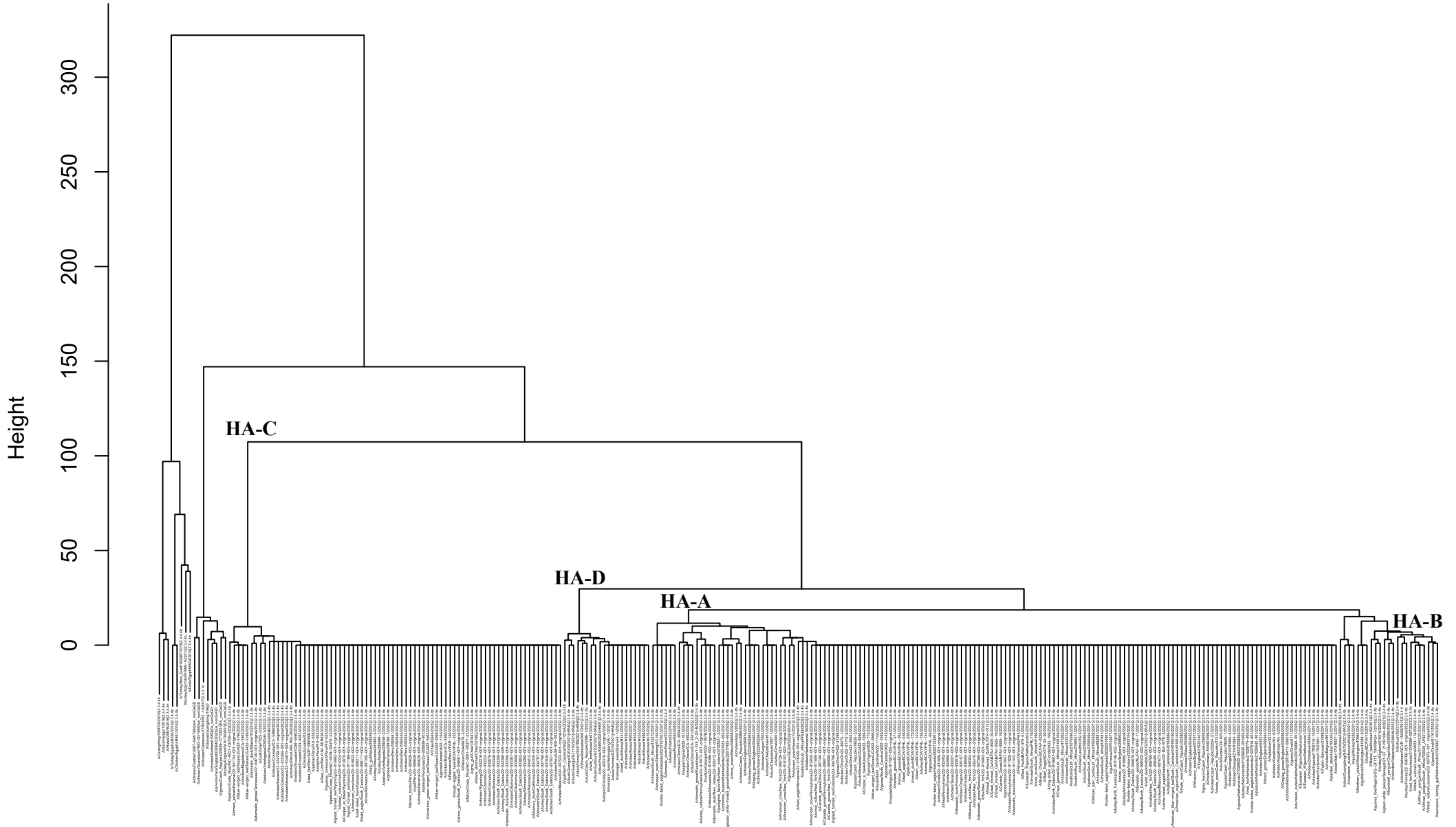

Supplement: SUPPLEMENTARY DATA SHEET 4 — Clustering analyses according to epitope variation. Predicted clusters are shown. [file Data_Sheet_4.PDF]
