# Supplementary material for: Sequence-based epitope mapping of high pathogenicity avian influenza H5 clade 2.3.4.4b in Latin America
Source: Front Vet Sci. 2024 Apr 29;11:1347509. doi: 10.3389/fvets.2024.1347509 (PMC11091830; doi:10.3389/fvets.2024.1347509)
Supplement: SUPPLEMENTARY DATA SHEET 1 — Phylogenetic trees of HA and NA genes. Labels in bold highlight strains of the panzootic outbreak detected in America. Name case in green correspond to genes from Central American and in red to genes from South American strains. Branches are colored according to their HA phylogenetic origin. Blue: American; Green: European Non-goose/Guangdong HPAI. Brown: Goose/Guangdong Lineage; Purple: 2.3.4.4b clade; Red: American 2.3.4.4b sub-clade. For NA, red branches correspond to genes from strains of the H5N1 2.3.4.4b clade detected in America. The proposed subclades into the clades are shown. [file Data_Sheet_1.PDF]

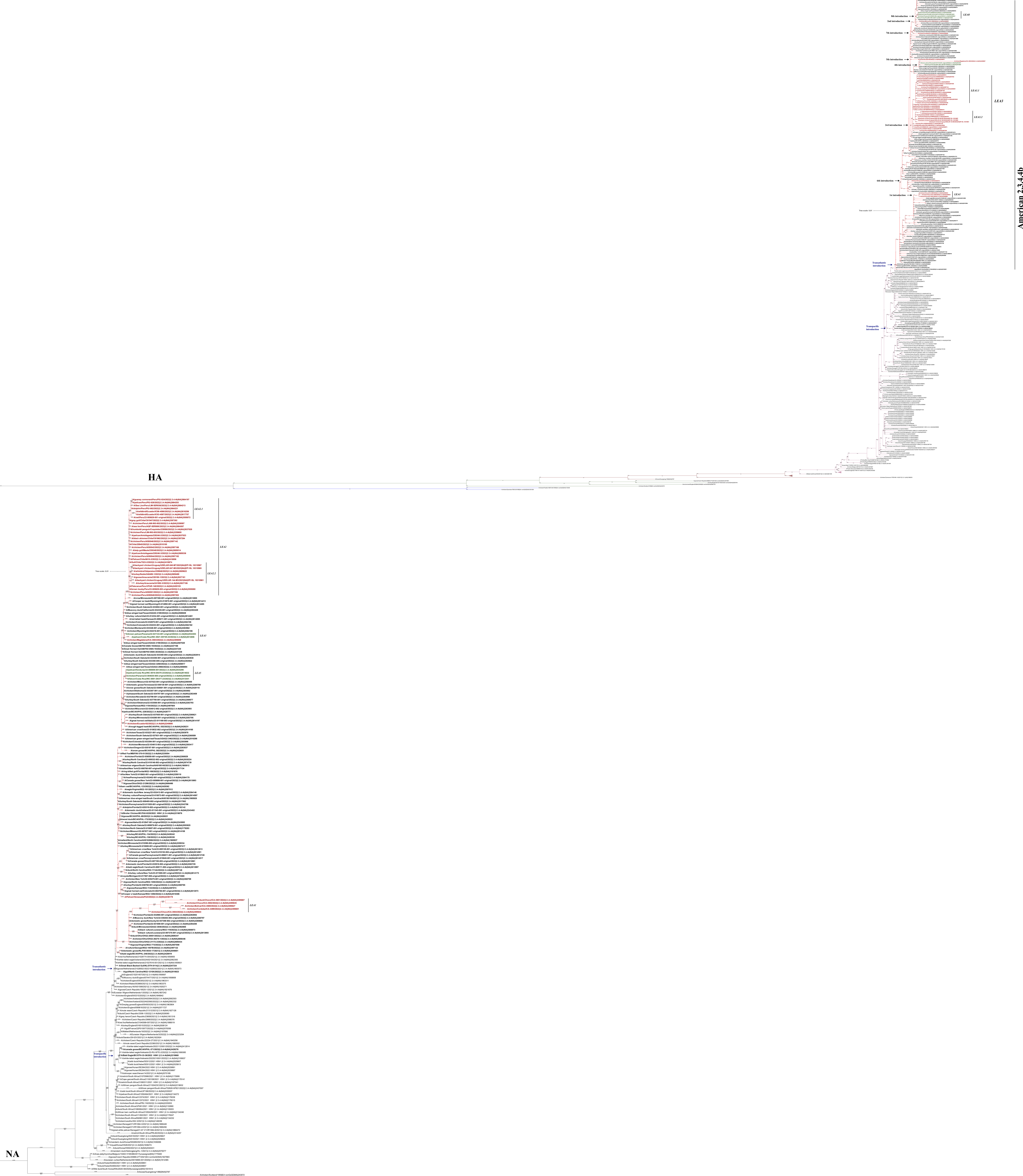
HA
NA
American 2.3.4.4b
LEA1
LEA2
LEA2.1
LEA2.2
LEA3
LEA3.1
LEA3.2
LEA4
LEA5
1st introduction
2nd introduction
3rd introduction
4th introduction
5th introduction
6th introduction
7th introduction
8th introduction
9th introduction
Transatlantic introduction
Transpacific introduction
Tree scale: 0.01
