# Supplementary material for: Sequence-based epitope mapping of high pathogenicity avian influenza H5 clade 2.3.4.4b in Latin America
Source: Front Vet Sci. 2024 Apr 29;11:1347509. doi: 10.3389/fvets.2024.1347509 (PMC11091830; doi:10.3389/fvets.2024.1347509)
Supplement: SUPPLEMENTARY DATA SHEET 2 — Phylogenetic trees of internal genes. Branches are colored according to their phylogeny. Blue: American; Red: Eurasian. Labels in bold highlight strains detected in America. Viruses labeled in green and red came from Central and South America, respectively. The proposed sub-clades into the clades are shown. [file Data_Sheet_2.PDF]

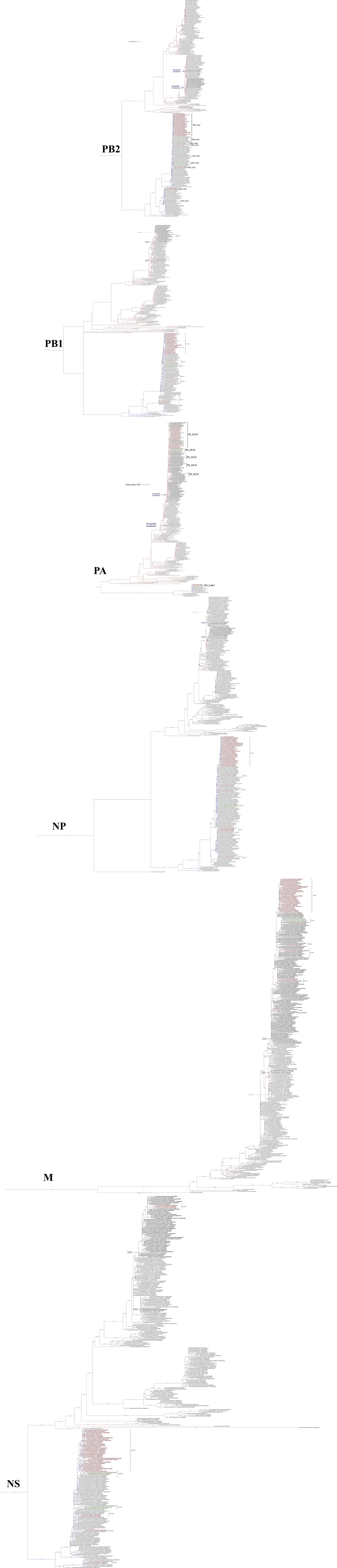
PB2
PB1
PA_LEA3
PA_LEA6
PA_LEA2
PA_LEA4
PA_LEA5
Tree scale: 0.01
Transatlantic introduction
Transpacific introduction
PA
NP
M
NS
